# Rightward dominance in temporal high-frequency electrical asymmetry corresponds to higher resting heart rate and lower baroreflex sensitivity in a heterogeneous population

**DOI:** 10.1002/brb3.343

**Published:** 2015-05-01

**Authors:** Charles H Tegeler, Hossam A Shaltout, Catherine L Tegeler, Lee Gerdes, Sung W Lee

**Affiliations:** 1 Department of Neurology, Wake Forest School of Medicine (WFSM)Winston-Salem, North Carolina; 2 Hypertension and Vascular Research Center, Department of Obstetrics and Gynecology, WFSMWinston-Salem, North Carolina; 3 Brain State Technologies, LLCScottsdale, Arizona

**Keywords:** Autonomic nervous system, heart rate variability, hemispheric asymmetry, neurotechnology, RDoC, temporal lobe

## Abstract

**Objective:**

Explore potential use of a temporal lobe electrical asymmetry score to discriminate between sympathetic and parasympathetic tendencies in autonomic cardiovascular regulation.

**Methods:**

131 individuals (82 women, mean age 43.1, range 13–83) with diverse clinical conditions completed inventories for depressive (CES-D or BDI-II) and insomnia-related (ISI) symptomatology, and underwent five-minute recordings of heart rate and blood pressure, allowing calculation of heart rate variability and baroreflex sensitivity (BRS), followed by one-minute, two-channel, eyes-closed scalp recordings of brain electrical activity. A temporal lobe high-frequency (23–36 Hz) electrical asymmetry score was calculated for each subject by subtracting the average amplitude in the left temporal region from amplitude in the right temporal region, and dividing by the lesser of the two.

**Results:**

Depressive and insomnia-related symptomatology exceeding clinical threshold levels were reported by 48% and 50% of subjects, respectively. Using a cutoff value of 5% or greater to define temporal high-frequency asymmetry, subjects with leftward compared to rightward asymmetry were more likely to report use of a sedative-hypnotic medication (42% vs. 22%, *P* = 0.02). Among subjects with asymmetry of 5% or greater to 30% or greater, those with rightward compared to leftward temporal high-frequency asymmetry had higher resting heart rate (≥5% asymmetry, 72.3 vs. 63.8, *P* = 0.004; ≥10%, 71.5 vs. 63.0, *P* = 0.01; ≥20%, 72.2 vs. 64.2, *P* = 0.05; ≥30%, 71.4 vs. 64.6, *P* = 0.05). Subjects with larger degrees of rightward compared to leftward temporal high-frequency asymmetry had lower baroreflex sensitivity (≥40% asymmetry, 10.6 vs. 16.4, *P* = 0.03; ≥50% asymmetry, 10.4 vs. 16.7, *P* = 0.05).

**Conclusion:**

In a heterogeneous population, individuals with rightward compared to leftward temporal high-frequency electrical asymmetry had higher resting heart rate and lower BRS. Two-channel recording of brain electrical activity from bilateral temporal regions appears to hold promise for further investigation as a means to assess cortical activity associated with autonomic cardiovascular regulation.

## Introduction

In the economically developed world, dysregulation of the autonomic nervous system (ANS) is associated with numerous disorders of health and behavior, and it is a major underlying basis for many needs in health care and even social services (Rees 2014). For example, pharmacological blockade of beta receptors associated with the sympathetic nervous system has been the cause of a “revolution in human pharmacotherapeutics” (Frishman 2013), and virtually any intervention for the cardiovascular system can be considered to have autonomic modulation as a partial if not primary objective. From the broadest perspective, the link between ANS dysregulation and health disorders may be an expression of evolutionary mismatch, a concept which includes the idea that much of modern disease may stem from chronically maladaptive functionality in stress response systems that evolved in a very different environment (Yun et al. 2004).

As yet, the greater potential for appreciation of the ANS either to inform health assessment, to serve as a basis for advanced interventions, or both, is still likely unrealized. One obstacle to advancing an ANS perspective may be the absence of a clinically viable, point-of-care approach to assessing key underlying mechanisms that drive an individual's tendencies for autonomic regulation. Heart rate variability (HRV) is one established metric of the capacity of the ANS to make fine-tuned adjustments to its functionality (Thayer et al. 2012) such that organ system demands can be met and anticipated in a flexible and highly calibrated manner. A wealth of literature exists on the capacity of HRV to stratify risk for adverse health outcomes related to the cardiovascular system (Huikuri and Stein 2013), and studies have also reported impaired HRV in neurological, psychiatric, and metabolic disorders (Collins et al. 2012; Kemp and Quintana 2013; Stuckey and Petrella 2013) among others.

Because autonomic regulation of the heart and other organs is under management from upstream sources (Saper 2002), the present study aimed to explore whether understanding and clinical practice related to ANS regulation may be advanced by a simple technique focused on the brain. To this end, we considered that hemispheric lateralization of autonomic management might potentially be leveraged to assay the brain–heart axis. Studies have found that the right and left hemispheres are predominantly engaged with management of the sympathetic and parasympathetic divisions, respectively (Zamrini et al. 1990; Yoon et al. 1997; Wittling et al. 1998; Hilz et al. 2001; Avnon et al. 2004), and this specialization may be related to activity of bilateral insular cortices (Oppenheimer et al. 1992). We hypothesized that, in a cross-sectional study of a clinically heterogeneous population, values for a high-frequency electrical asymmetry score calculated on the basis of the noninvasive temporal scalp recordings would correspond to differences in peripherally measured autonomic cardiovascular regulation, with rightward or leftward dominance being indicative of relative sympathetic or parasympathetic activation, respectively.

## Methods

Data were collected from a cohort of individuals who provided informed consent, and consecutively enrolled in a prospective, open label, IRB-approved study in the Department of Neurology at Wake Forest School of Medicine. The project evaluated the use of a noninvasive, closed-loop, acoustic stimulation neurotechnology known as High-resolution, relational, resonance-based, electroencephalic mirroring (HIRREM®) by individuals with diverse neurological, cardiovascular, and psychophysiological conditions. Potential participants were identified by referral, based on the presence of an underlying neurological, cardiovascular, or psychophysiological disorder, and were enrolled to collect feasibility pilot data across a variety of conditions, as well as to assess effect size. Exclusion criteria included inability to provide informed consent, or attend the study visits, known seizure disorder, bilateral severe hearing impairment, a need for ongoing use of recreational drugs, or a need for ongoing treatment with opiates, benzodiazepines, or antipsychotic medications, except as deemed acceptable by the principal investigator. Baseline assessments included self-reporting of medication or supplement use (without asking the date or time of last usage), and completion of self-report inventories for evaluation of depressive symptoms, using either the Center for Epidemiologic Studies Depression Scale (CES-D; Radloff 1977) or the Beck Depression Inventory-II (BDI-II; Beck et al. 1996), and sleep-related symptomatology, using the Insomnia Severity Index (ISI; Morin et al. 1993).

Short-term heart rate and blood pressure recordings (5 min, 1000 Hz, BIOPAC, Santa Barbara, CA) were obtained during spontaneous breathing, while participants rested in a supine position. Heart rate (HR, beats/min) and heart rate variability (HRV) measures in time (standard deviation of the normal-to-normal interval, SDNN) and spectral high-frequency (HF, 0.15–0.40 Hz) domains as well as baroreflex sensitivity (BRS, msec/mmHg), using the sequence method, were calculated using software for analysis of HRV and BRS (Nevrokard BRS**,** Medistar, Ljubljana, Slovenia). SDNN, HF, and BRS are, respectively, an index of beat-to-beat variability in the heart period, a spectral analysis measure of variability in the range of 0.15–0.40 Hz, and a measure of changes in the R to R interval that follow changes in systolic blood pressure. SDNN, HF, and BRS are generally understood to be predominantly indicative of parasympathetic activity, whereas resting heart rate reflects a mixture of sympathetic and parasympathetic influences (Task Force 1996; La Rovere et al. 2008).

**Figure 1:**
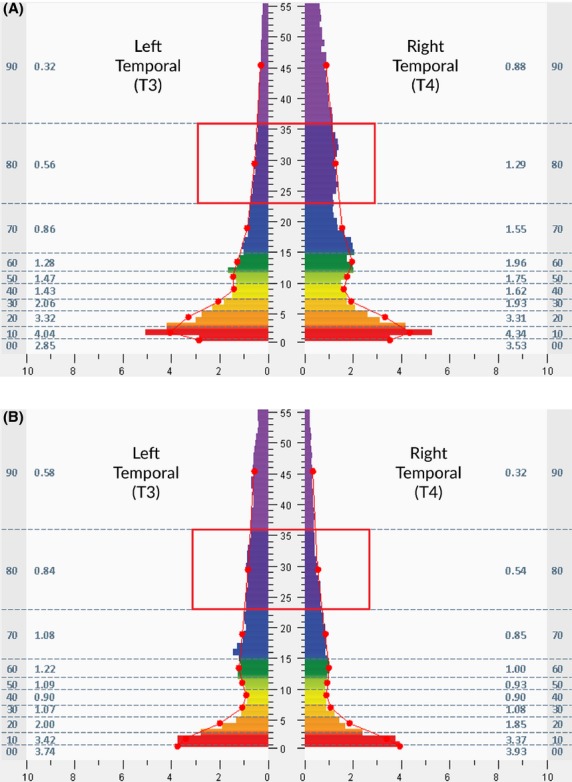
(A and B) FFT spectral displays, as examples of observed electrical brain activity data from two study participants. Frequency (Hz, central *Y* axis) is plotted against transformed amplitude (μv, *X* axis). Data represent one minute of data recorded from the right (T4) and left (T3) temporal montage with eyes closed at the baseline assessment. Red boxes denote the high frequency, 23–36 Hz range analyzed for temporal high-frequency electrical asymmetry. (A) is from a 14-year-old male athlete who enrolled for persisting postconcussion symptoms, primarily headache [note rightward asymmetry in the high frequencies (red box)], while (B) is from a 57-year-old female who reported a variety of symptoms, but enrolled primarily for insomnia [note leftward asymmetry in high frequencies (red box)].

Within 30 minutes after completion of the heart rate and blood pressure recording, subjects relocated to a different room to undergo an assessment of brain electrical activity using the technique of a standard HIRREM assessment (Gerdes et al. 2013). Subjects were seated upright and asked to relax while undergoing two-channel scalp recordings at primarily homologous hemispheric regions. Brain electrical activity data was recorded from at least six different locations (F3F4, C3C4, P3P4, T3T4, FZOZ, and O1O2, in the 10–20 International EEG system), 3 min epochs for each location (one minute eyes closed, one minute eyes partially closed, and one minute eyes open) and subjected to automated time domain analysis. For purposes of the present report, only the one minute of data from the eyes-closed T3T4 recording was subject to further analysis. A high-frequency (23–36 Hz) band was selected and filtered as the range of interest for analysis (Fig.1), on the basis that activity in this range may be taken as an indication of cortical activation (Amzica and Lopes da Silva 2011). Electrical amplitudes (microvolts) in this range were aggregated as a high-frequency band average. The HIRREM approach is designed to be insensitive to recording artifacts (Gerdes et al. 2013). To be consistent with the procedural needs of a point-of-care intervention in a resource-sensitive context, no attempt was made to identify sub-epochs of data that may have reflected noncortical factors (e.g., eye blinks or muscular contractions). A temporal lobe high-frequency electrical asymmetry percentage score was calculated for each subject by subtracting the value for the high-frequency band average at T3 from the value at T4 and dividing by the lesser of the two, yielding a positive score for rightward (T4) asymmetry.

Chi-square tests were used to compare leftward versus rightward dominant subjects with respect to self-reported use of medications, in categories based on their prevalence of use as well as known effects on autonomic regulation. T-tests were used to compare measures of autonomic cardiovascular regulation for subjects with rightward versus leftward dominant temporal high-frequency electrical asymmetry, using arbitrarily chosen values ranging from 5% to 50% to indicate dominant asymmetry. Statistical analyses were performed using the Statistical Package for the Social Sciences (SPSS; IBM SPSS Statistics for Windows, Version 22.0.), IBM Corporation, Armonk, NY.

## Results

Results are reported for 131 individuals (82 women, mean age 43.1, range 13–83) for whom complete autonomic and brain electrical activity data were available. Subjects' primary health concerns on study enrollment were insomnia (22%), traumatic brain injury or concussion (15%), hot flashes (12%), posttraumatic stress disorder (11%), headaches or migraine (7%), postural orthostatic tachycardia syndrome (5%), or other neurological or neuropsychiatric conditions including attention deficit disorder, Asperger syndrome, symptoms related to prior cancer treatment, and others (27%). For many subjects, scores met or exceeded clinical thresholds for depressive mood (CES-D ≥16 or BDI-II ≥20, 48%) and moderate to severe insomnia (ISI ≥15, 50%). Self-reported medication or supplement use is shown in Table 1, for subjects who had either rightward or leftward asymmetry of 5% or greater.

**Table 1:** Percentage of subjects using various medications or supplements, listed according to their baseline dominance pattern in temporal high-frequency asymmetry. *P* values  ≤ 0.05 are indicated with an asterisk

	≥5% Leftward Dominant (*n* = 45)	≥5% Rightward Dominant (*n* = 64)	*P*-value for difference
Beta-Blocker	13.3	10.9	0.71
Antidepressant	22.2	14.1	0.27
Sedative-hypnotic	42.2	21.9	0.02^*^
Other neuropharmacological agent	17.8	12.5	0.45
Statin	13.3	7.8	0.35
Herb or supplement with known effects on ANS	35.6	32.8	0.77
Thyroid medication	11.1	7.8	0.56
ACE-I or ARB	6.7	6.3	0.93
Stimulant	6.7	6.3	0.93
Other hormone	15.6	18.8	0.67
Diabetes-related medication	0	3.1	0.23
Calcium channel blocker	4.4	7.8	0.49

Table 2 shows averages for metrics of autonomic cardiovascular regulation for subjects who demonstrated rightward (T4) or leftward (T3) temporal high-frequency electrical asymmetry. Among subjects with asymmetry of 5% or greater to 30% or greater, rightward dominance was associated with higher resting heart rate. Removal of data of two outliers who had rightward dominant asymmetry and very high heart rates (122 and 160 beats per minute) did not alter the main findings. Among those subjects with asymmetry of 40% or greater or 50% or greater, rightward dominance was associated with lower BRS. Across all the cutoff points evaluated for defining asymmetry, those with leftward dominance had higher values for HRV as measured in the time domain (SDNN) and in the HF spectral domain, when compared to those with rightward dominance, although these differences were not statistically significant.

**Table 2:** Averages for metrics of autonomic cardiovascular regulation for a heterogeneous clinical population (*n* = 131), for subjects with rightward versus leftward dominance of temporal high frequency electrical activity, using a range of values for defining asymmetry. Metrics analyzed include heart rate, the standard deviation of all normal RR intervals (SDNN), spectral high frequency (HF), and baroreflex sensitivity (BRS). *P* values ≤0.05 are indicated with an asterisk

	Heart rate (beats/min)	SDNN (ms)	HF (ms^2^)	BRS (ms/mmHg)
≥5% asymmetry				
T3 group average (*n* = 45)	63.8	46.0	971.3	14.8
T4 group average (*n* = 64)	72.3	39.6	620.7	13.0
*P* value	0.004^*^	0.09	0.16	0.32
≥10% asymmetry				
T3 group average (*n* = 36)	63.0	46.2	1098.8	14.6
T4 group average (*n* = 51)	71.5	41.3	694.0	14.2
*P* value	0.01^*^	0.27	0.18	0.86
≥20% asymmetry				
T3 group average (*n* = 30)	64.2	46.4	1141.9	14.3
T4 group average (*n* = 42)	72.2	42.4	765.3	14.7
*P* value	0.05^*^	0.44	0.30	0.89
≥30% asymmetry				
T3 group average (*n* = 25)	64.6	46.7	1022.7	15.2
T4 group average (*n* = 33)	71.4	41.1	718.3	14.0
*P* value	0.05^*^	0.32	0.44	0.68
≥40% asymmetry				
T3 group average (*n* = 20)	63.5	46.2	948.0	16.4
T4 group average (*n* = 23)	69.1	37.2	473.9	10.6
*P* value	0.09	0.17	0.21	0.03^*^
≥50% asymmetry				
T3 group average (*n* = 16)	64.2	46.2	790.9	16.7
T4 group average (*n* = 19)	67.5	35.3	435.6	10.4
*P* value	0.37	0.13	0.33	0.05^*^

## Discussion

In a study of subjects with heterogeneous conditions who were enrolled in a prospective study to evaluate a noninvasive acoustic stimulation neurotechnology, we compared baseline values for measures of autonomic cardiovascular regulation in those subjects with either rightward or leftward hemispheric asymmetry of temporal high-frequency electrical activity. Using scores from 5% or greater, to 30% or greater, to define asymmetry, subjects with rightward temporal dominance manifested higher resting heart rate. Using scores from 40% or greater, or 50% or greater, to define asymmetry, those with rightward temporal dominance had lower BRS. These findings provide partial support for the hypothesis that rightward or leftward dominance in a temporal high-frequency electrical asymmetry score, collected through a simple and rapid approach, may indicate relative dominance of sympathetic or parasympathetic activity, respectively, in autonomic cardiovascular regulation.

The present data build on other studies that have reported correspondences between asymmetry in scalp-recorded temporal lobe activity and autonomic regulation or cardiovascular function. Foster and colleagues showed that rightward asymmetry of temporal lobe brain electrical activity discerned through surface measures correlated with blood pressure (2006) and to a lesser degree with heart rate (2006; 2008) in healthy young adults. Gray et al. (2007) showed that in a group of male patients with cardiovascular disease managed with medications, greater negativity of a heartbeat-associated cortical potential in the left temporal region was associated with increased cardiac output and increased cardiac repolarization inhomogeneity. This report extends those findings in a more heterogeneous sample, using a simple technique (one minute, two-channel recording of brain electrical activity, without de-artifacting), by suggesting an association between rightward temporal dominance and lower BRS.

Our findings could be interpreted to suggest, as others have reported (Gray et al. 2007), that a noninvasive measure of asymmetrical temporal activity may reflect afferent signaling from the heart, that both cortical asymmetry and autonomic regulation patterns are joint expressions of an underlying autonomic genotype, or that efferent neural regulation of the heart has representation in the cortex. Future studies might be designed to discriminate among these explanations. Notably the latter of these possibilities would have interventional implications for autonomic modulation, on the basis that attention to asymmetry in hemispheric activity may be a way to advance the objective of more optimal top-down neurogenic regulation of the heart, gut, and other systems (Samuels 2007; Furness 2012; Goldstein 2012; Sterling 2012; Lee et al. 2014). This strategy has been used to explore direct stimulation of the left temporal lobe as a way to modify parasympathetic withdrawal and thereby enhance performance in cyclists, for example (Okano et al. 2013). In exploratory analysis of longitudinal data from the population enrolled in the present study, we have reported that use of a noninvasive closed-loop neurotechnology was associated with reduction of temporal high-frequency asymmetry and increased HRV (Tegeler et al. 2013).

In larger context, the present study appears to support the objectives of the Research Domain Criteria (RDoC; Cuthbert and Insel 2013; Cuthbert 2014) of the National Institutes of Mental Health (NIMH). The RDoC framework is intended to advance mental and behavioral health sciences by organizing research in these fields on the foundation of core modules or domains of brain-behavior functionality that have salience in both health and disease, and across disease states. Arousal, to include autonomic regulation, is one of the five core modules (along with positive valence, negative valence, cognitive systems, and systems for social processes) that have been recognized by RDoC working groups. The RDoC framework is associated with a matrix of units of analysis from the level of genes to self-reports, and we propose that temporal asymmetry merits further investigation for inclusion in this matrix as a physiological unit of analysis for the domain of arousal. Elsewhere we have commented (Tegeler et al. 2014) that this usage is consistent with a recent PET study which found that rightward dominant activity in a temporal lobe structure (right anterior insula) represented a treatment response biomarker for patients with major depression (McGrath et al. 2013).

The lower rate of reported use of sedative-hypnotic medications by individuals with rightward compared to leftward dominance may be explained in ways that are consistent with temporal high-frequency asymmetry being an indicator of arousal. Use of sedative-hypnotics may have represented pharmacological modulation of sympathetic tone that was associated with attenuation of rightward temporal dominance. Or, for any given individual not taking medications that modulate arousal, rightward temporal dominance (sympathetic or “fight or flight” tendencies) may have been less subjectively tolerable than leftward dominance (parasympathetic or “rest, digest, or freeze” tendencies), such that unmedicated rightward dominant individuals may have been more motivated to enroll in the study, than were unmedicated individuals with leftward dominance.

Several aspects of the present study must be noted as considerations or limitations. As a matter of principle, enrollment of study subjects across a range of diagnostic categories is consistent with the RDoC approach, in that such a strategy permits recognition of biological phenomena that cut across those categories (Cuthbert and Insel 2013; Cuthbert 2014). Nonetheless the clinical heterogeneity and wide age range of the subjects raise the question of whether the relationship being reported is related to specific characteristics of the study population. Subjects reported using a variety of medications and supplements that have known effects on autonomic functioning, and we did not attempt to confirm the specific date or time of their last usage. Exploratory analysis did not suggest that our findings were driven by subjects' age, gender, clinical diagnoses, or self-reported sedative-hypnotic use (data not shown). Cardiovascular and brain electrical activity data were not collected simultaneously, and it can be presumed that subjects underwent at least some degree of transition in their neurally directed autonomic states, from the time they completed their heart rate and blood pressure recordings to the time they underwent their brain activity recording (in a different room). While we recognize the likely and nontrivial influence of all the above variables, we would expect their impact on the analysis to be in the direction of attenuation or nullification of the main findings. Future studies may aim to examine relationships between temporal asymmetry and autonomic cardiovascular regulation in more homogeneous populations. Additionally, determination of a relationship between temporal electrical asymmetry and autonomic regulation should benefit from advanced analytic strategies that account for activity in other frequency ranges, as well as possible influence from eyes being closed or open.

It is important to note that our analysis strategy for the main results compared individuals with rightward versus leftward asymmetry, thereby excluding individuals who had relatively lower asymmetry scores by absolute value. An initial analysis that compared asymmetry to autonomic metrics for the entire cohort was not statistically significant. While it is possible that the present findings reflect a Type I error, we think it more likely that the absence of a correlation among individuals who were more symmetrical was related either to one or more of the attenuating factors listed above, or to a phenomenon wherein the true signal for this relationship is most readily demonstrated in individuals who have more marked asymmetry. For example, biologists have recognized that fluctuating asymmetry in a variety of morphological traits can be used to indicate environmental stress (Parsons 1990). It would seem plausible that the more asymmetrical subjects in our population were those who had accumulated a greater degree of life stress, which could in turn be associated with altered tendencies in autonomic regulation (Heim et al. 2000; Lee et al. 2014).

In conclusion, the present study found that in a cross-sectional analysis in a heterogeneous population enrolled in a prospective study of an acoustic stimulation neurotechnology, baseline rightward versus leftward dominant asymmetry in temporal lobe high-frequency electrical activity patterns was associated with higher resting heart rate and lower BRS. This study is the first to show that it may be possible to detect an altered level of autonomic cardiovascular regulation by using a noninvasive, one-minute, two-channel recording methodology. Noninvasive measurement of cortical electrical asymmetry merits further investigation as a potential means to index autonomic activity, and may represent a novel path for brain-centric, top-down strategies for supporting improved autonomic regulation.

## Conflict of Interest

Authors from the Wake Forest School of Medicine have no conflicts to report. Sung W. Lee and Lee Gerdes are employees of Brain State Technologies, LLC, developer of the neurotechnology used to collect brain electrical activity data reported in the study. This material is based upon work supported by a research grant from The Susanne Marcus Collins Foundation, Inc., to Wake Forest School of Medicine, and the Army STTR Program Office and the US Army Research Office under Contract No. W911NF-14-P-0047 with Brain State Technologies. Any opinions, findings, and conclusions or recommendations expressed in this material are those of the authors and do not necessarily reflect the views of the Army STTR Program or the US Army Research Office.
